# Safety and efficacy of robot-assisted total knee arthroplasty: a network meta-analysis and systematic review

**DOI:** 10.1186/s13018-026-06889-y

**Published:** 2026-05-02

**Authors:** Jixiong Yong, Han Li, Zhenhua Wu, Zhong Cao

**Affiliations:** Leshan Hospital of Traditional Chinese Medicine, Baiyang Road, Leshan, 614000 Sichuan Province China

**Keywords:** Robot-assisted, Total knee arthroplasty, Network meta-analysis, Systematic review

## Abstract

**Background:**

In the context of the aging of the global population, the prevalence of knee joint disorders continues to rise. Concurrently, the integration of robotic systems and intelligent implants represents an inevitable trend in orthopedic surgery. A comprehensive evaluation of the safety and effectiveness of robot-assisted total knee arthroplasty (RA-TKA) is therefore urgently needed to inform clinical decision-making.

**Objective:**

To explore the advantages of 9 RA-TKAs across 8 outcomes.

**Methods:**

A systematic literature search was conducted in the PubMed, Web of Science, Embase, Cochrane Library, CBM, CNKI, Wanfang, and VIP databases from inception to December 1, 2025. The risk of bias and methodological quality were assessed via Review Manager (version 5.4). Network meta-analysis was performed via RStudio (version 4.4.1).

**Results:**

A total of 36 studies involving 2841 patients were included. In direct comparisons, conventional TKA (C-TKA) yielded shorter operative times than MAKO, HURWA, SkyWalker, ROSA, and Brainlab Knee did. CORI also had a shorter operative time than Brainlab Knee did. Compared with the C-TKA, MAKO, HURWA, SkyWalker and TiRobot groups, the ROSA group presented higher KSS-knee scores. In addition, C-TKA, HURWA, and CORI presented higher KSS-knee scores than did SkyWalker. For the KSS-function scores, the C-TKA and ROSA scores were higher than the HURWA score. C-TKA demonstrated a greater postoperative ROM than HURWA did. For HKA angle deviation, C-TKA resulted in greater deviation than MAKO, HURWA, SkyWalker, TiRobot, and EPMEDBOT did. In the comprehensive best probability ranking, C-TKA (93%) ranked highest in terms of operative time. SkyWalker (87%) ranked highest in terms of blood loss. SkyWalker (91%) ranked highest in terms of the KSS-knee scores. HURWA (87%) ranked highest in terms of the KSS function scores. MAKO (85%) ranked highest for HSS. The YUANHUA (76%) ranked highest for the WOMAC. The CORI (69%) ranked highest for ROM. SkyWalker (87%) ranked highest for HKA angle deviation.

**Conclusions:**

Overall, RA-TKA demonstrated superior safety and effectiveness compared with C-TKA, with different robotic systems exhibiting distinct advantages across outcome measures. Nevertheless, C-TKA retains a significant advantage in reducing the operative time, highlighting an important area for further optimization of robotic-assisted TKA.

**Supplementary Information:**

The online version contains supplementary material available at 10.1186/s13018-026-06889-y.

## Introduction

Total knee arthroplasty (TKA) is a well-established and widely performed surgical procedure for the treatment of end-stage knee osteoarthritis (KOA), rheumatoid arthritis (RA), and other degenerative knee disorders [1–3]. Conventional TKA (C-TKA), first introduced in 1958, was initially developed for the treatment of varus deformity and medial compartment disease of the knee [4, 5]. In recent years, the global volume of TKA has increased substantially; in the United States alone, more than 1 million TKAs are performed annually, with projections exceeding 3 million procedures by 2040 [6–8]. However, despite continuous advancements in surgical techniques and implant design, C-TKA still has inherent limitations, which may result in postoperative malalignment, a restricted range of motion, and other complications [9–11].

The first orthopedic robotic-assisted system, ROBODOC, was developed in 1986 and was applied to cementless total hip arthroplasty (THA) in 1992. Early robot-assisted total knee arthroplasty (RA-TKA) systems were introduced in Europe in the late 1980s [12, 13]. RA-TKA systems primarily utilize computer software integrated with advanced imaging technologies to generate patient-specific virtual three-dimensional reconstructions of the knee joint [14–17]. Compared with C-TKA, RA-TKA has been associated with greater surgical accuracy, smaller incisions, and improved postoperative outcomes. However, the comparative advantages of different RA-TKA systems remain unclear [18–20]. At present, commonly used RA-TKA systems include MAKO, ROSA, CORI, and several systems independently developed in China, such as SkyWalker and TiRobot [21]. Therefore, to evaluate the comparative advantages of different RA-TKA systems, this study aims to systematically assess the safety and effectiveness of the MAKO, HURWA, SkyWalker, Yuanhua, CORI, ROSA, Brainlab Knee, TiRobot, and EPMEDBOT systems via network meta-analysis.

## Methods

### Literature search and eligibility criteria

A comprehensive literature search was conducted in the electronic databases PubMed, Web of Science, Embase, the Cochrane Library, CBM, CNKI, Wanfang, and VIP from inception to December 1, 2025. The search strategy used the keywords robot-assisted, total knee arthroplasty, randomized, random, and comparison, which were searched in all fields. The search was limited to studies published in English or Chinese.

Study selection was performed independently by two reviewers, with discrepancies resolved by a third reviewer. The inclusion criteria were as follows: (1) randomized controlled trials (RCTs) or retrospective cohort studies (RCs); (2) studies involving patients undergoing TKA; (3) studies reporting at least one of the following outcomes: operative time, blood loss, Knee Society score (KSS)-knee score, KSS-function score, Hospital for Special Surgery (HSS) score, Western Ontario and McMaster Universities Osteoarthritis Index (WOMAC) score, range of motion (ROM), and hip–knee–ankle (HKA) angle deviation; and (4) robot-assisted procedures involving preoperative planning, intraoperative positioning, bone resection, trial implantation, and prosthesis implantation.

The exclusion criteria were (1) non-RCT and nonretrospective study designs, (2) failure to report any of the predefined outcomes, (3) incomplete or unavailable data, (4) publications in languages other than English or Chinese, and (5) duplicate studies.

### Data extraction and quality assessment

Two researchers (Zhenhua Wu and Han Li) independently screened and verified the eligible studies, with disagreements adjudicated by a third researcher (Jixiong Yong). The screening process included the removal of duplicate records, initial screening of titles, evaluation of abstracts and keywords, and full-text review to ensure data completeness. The following information was extracted: first author, year of publication, country, sample size (male/female), mean age, follow-up duration, intervention details, and outcome measures.

The risk of bias in the included studies was assessed via Review Manager 5.4 [22]. evaluating random sequence generation, allocation concealment, blinding, completeness of outcome data, selective reporting, and other potential sources of bias.

### Statistical analysis

Network meta-analysis was performed via RStudio (version 4.4.1) with the “gemtc” package. All outcomes were treated as continuous variables and analyzed via mean differences (MDs) with corresponding 95% confidence intervals (CIs) via a random effects model. Pairwise comparisons and optimal ranking probabilities were generated. Model convergence was assessed via Brooks–Gelman–Rubin diagnostic plots and the potential scale reduction factor (PSRF). Consistency within closed loops of the network was evaluated via the node-splitting method [23]. Bayesian Markov chain Monte Carlo (MCMC) simulations were conducted via JAGS via the “rjags” package. Four chains were run with initial values set at 2.5, a burn-in of 5,000 iterations, and a total of 10,000 iterations for parameter estimation [24].

## Results

### Literature search results

**Fig. 1:**
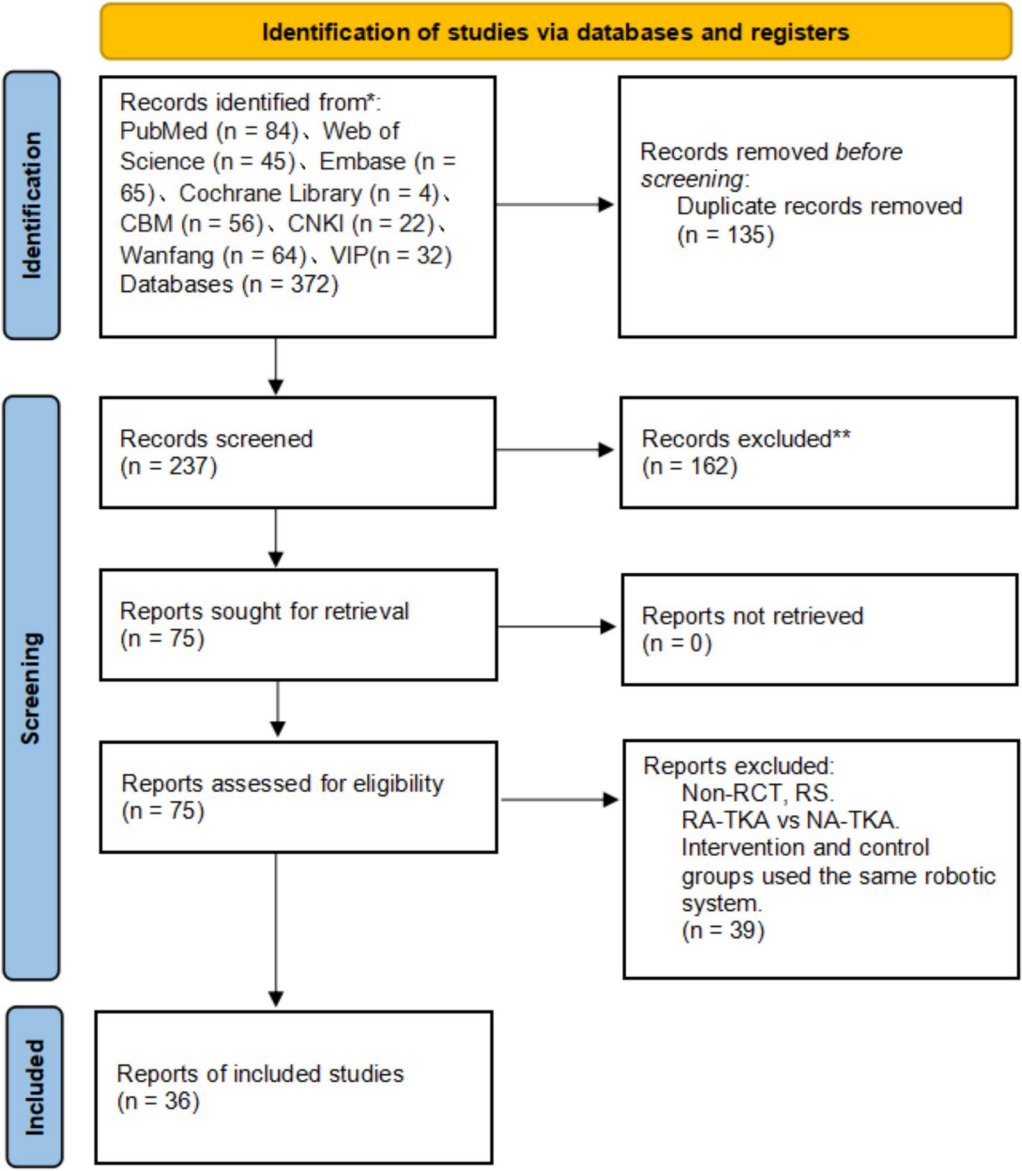
Flow chart of PRISMA

A total of 372 studies were initially identified through the literature search. After the removal of duplicate records, screening of titles and abstracts, and full-text assessment for eligibility, 36 studies [25–60] met the inclusion criteria and were included in the network meta-analysis. The study selection process is illustrated in Fig. 1.

Study characteristics.

The basic characteristics of the included studies included the first author, year of publication, country, sample size (male/female), mean age, interventions, and outcomes. One study [60] included 2 comparison groups. The detailed study characteristics are summarized in Table 1.

**Table 1:** Basic information of the studies

Study	Countries	Sample size (Male/Female)		Mean age		Interventions		Outcomes
		T	C	T	C	T	C	
Chen M 2025 [25]	CN	9/6	7/8	64.3 ± 6.2	64.6 ± 7.3	CORI	C-TKA	(1)(2)(3)(4)
Xuefeng L 2025 [26]	CN	10/54	15/153	68.4 ± 6.3	69.3 ± 7.9	YUANHUA	C-TKA	(1)(2)
Wei W 2021 [27]	CN	3/8	2/11	64.9 ± 12.0	64.9 ± 12.0	HURWA	C-TKA	(1)(3)(4)(5) (6)
Zi’an Z 2022 [28]	CN	4/12	5/11	59.3 ± 8.3	60.6 ± 8.4	MAKO	C-TKA	(1)(2)(5)(7)
Ruoyu W 2024 [29]	CN	8/23	5/28	68.0 ± 4.7	69.2 ± 4.4	MAKO	C-TKA	(1)(3)(6)(8)
Zhonghua X 2022 [30]	CN	3/14	3/13	66.6 ± 3.7	67.3 ± 3.5	YUANHUA	C-TKA	(1)(2)(5)(6)
Xin Z 2023 [31]	CN	8/20	5/17	65.5 ± 5.8	65.2 ± 5.9	SkyWalker	C-TKA	(1)
Gang L 2023 [32]	CN	10/44	20/102	66.2 ± 6.4	66.8 ± 7.2	MAKO	C-TKA	(1)(2)(3)(4) (7)(8)
Hongping W 2024 [33]	CN	18/35	16/24	64.4 ± 8.5	64.3 ± 7.0	HURWA	Brainlab Knee	(1)(2)(3)(4) (7)(8)
Fan W 2023 [34]	CN	5/17	7/16	60.55 ± 5.61	59.78 ± 5.40	ROSA	C-TKA	(1)(3)(4)(5) (7)(8)
Qiaoqiao M 2024 [35]	CN	11/14	11/14	64.7 ± 14.8	64.7 ± 14.8	MAKO	C-TKA	(1)(2)(7)(8)
Haoyang L 2025 [36]	CN	21/45	18/41	68.44 ± 8.23	68.27 ± 6.44	MAKO	C-TKA	(1)(2)
Yang Y 2023 [37]	CN	10/13	8/15	67.54 ± 12.32	70.34 ± 9.74	HURWA	C-TKA	(1)(2)(3)(7) (8)
Wei L 2025 [38]	CN	5/16	6/16	67.0 ± 5.7	68.6 ± 6.0	HURWA	C-TKA	(1)(2)(3)(5) (6)(7)(8)
Mingyou W 2025 [39]	CN	15/50	12/53	64.6 ± 7.8	65.7 ± 10.2	HURWA	C-TKA	(1)(3)(6)(7) (8)
Xin Y 2024 [40]	CN	8/20	11/20	70.68 ± 5.17	71.39 ± 4.5	SkyWalker	C-TKA	(1)(2)(3)(4) (5)(6)(7)(8)
Shiliang Q 2025 [41]	CN	7/24	8/19	68.19 ± 8.58	67.06 ± 7.41	TiRobot	C-TKA	(1)(2)(3)(7) (8)
Zengbing X 2023 [42]	CN	10/6	7/9	68.05 ± 11.28	66.87 ± 10.39	HURWA	C-TKA	(1)(2)(3)(6) (8)
Mincong D 2023 [43]	CN	3/8	1/11	68.7 ± 7.8	70.8 ± 5.0	TiRobot	C-TKA	(1)(3)(7)(8)
Mingyou W 2025 [44]	CN	16/26	14/28	64.70 ± 8.30	65.60 ± 7.50	HURWA	CORI	(1)(2)(3)(4) (7)
Chunning H 2025 [45]	CN	3/47	6/41	66.58 ± 6.76	67.55 ± 7.8	HURWA	C-TKA	(1)(6)(8)
Haoming A 2023 [46]	CN	8/19	6/21	63.21 ± 3.76	66.34 ± 4.27	SkyWalker	C-TKA	(1)(2)
Jialiang H 2025 [47]	CN	6/19	5/25	65.20 ± 1.93	68.73 ± 1.36	ROSA	C-TKA	(1)(2)(3)(4) (7)(8)
Gang L 2023 [48]	CN	16/59	24/129	65.8 ± 6.8	66.9 ± 7.4	MAKO	C-TKA	(1)(3)(4)(7)
Qiaoqiao M 2025 [49]	CN	20/53	25/61	63.7 ± 5.8	64.1 ± 6.5	MAKO	C-TKA	(1)(2)(7)(8)
Zuhai H 2025 [50]	CN	8/15	10/13	59.43 ± 8.22	59.17 ± 8.03	MAKO	C-TKA	(1)(2)(5)(7) (8)
Genxiang R 2022 [51]	CN	3/6	3/6	68.22 ± 6.12	70.00 ± 10.63	HURWA	C-TKA	(1)(2)(6)(7) (8)
Xin Y 2025 [52]	CN	9/19	10/21	70.54 ± 4.80	74.39 ± 3.10	SkyWalker	C-TKA	(1)(2)(3)(8)
Rui H 2022[53]	CN	5/25	6/24	66.1	63.2	SkyWalker	C-TKA	(1)(2)(6)(8)
Mingcheng Y 2021 [54]	CN	9/19	4/28	65.2	65.4	YUANHUA	C-TKA	(3)(6)(7)(8)
Gaopeng D 2021 [55]	CN	6/13	3/18	67.21 ± 5.15	68.67 ± 4.86	HURWA	C-TKA	(5)(8)
Ma N 2024 [56]	CN	7/15	11/15	68.68 ± 7.92	66.15 ± 6.56	MAKO	C-TKA	(1)(8)
An H 2023 [57]	CN	7/20	5/22	65.81 ± 5.60	64.56 ± 6.87	SkyWalker	C-TKA	(1)(2)
Li Z 2022 [58]	CN	13/60	15/62	68.0 ± 7.97	69.0 ± 6.00	HURWA	C-TKA	(3)(5)
Ren YM 2025 [59]	CN	6/26	12/22	66.8 ± 5.8	66.8 ± 5.1	EPMEDBOT	C-TKA	(6)(8)
Feng S 2025 [60]	CN	7/36 8/33	19/49 10/56	64.9 ± 6.5	65.1 ± 5.7	TiRobot	C-TKA	(5)(6)(7)(8)

T: treatment group; C: control group; (1) operative time; (2) blood loss; (3) KSS-knee score; (4) KSS-function score; (5) HSS; (6) WOMAC; (7) ROM; (8) HKA angle deviation

### Risk of bias assessment

**Fig. 2:**
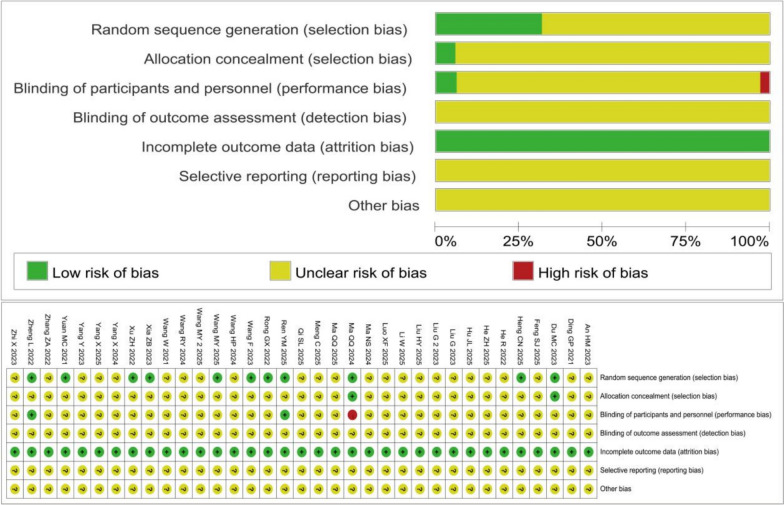
Risk of bias assessment proportion plot

In the risk of bias assessment, only 1 study [35] reported the absence of blinding and was therefore judged to be at high risk of bias. The remaining studies were assessed as having a low or moderate risk of bias. Two studies [35, 43] reported the use of envelope randomization with allocation concealment, and 2 studies [58, 59] employed a single-blind design. The overall risk of bias assessment is presented in Fig. 2.

### Network meta-analysis

For the operative time network, 31 studies [25–53, 56, 57] involving 2307 patients were included. Direct comparisons mainly involved MAKO, HURWA, SkyWalker, YUANHUA, CORI, ROSA, Brainlab Knee, and TiRobot versus C-TKA, with additional comparisons between HURWA and CORI and between HURWA and Brainlab Knee, enabling indirect comparisons among all robotic systems.

With respect to blood loss, 22 studies [25, 26, 28, 30, 32, 33, 35–38, 40–42, 44, 46, 47, 49–53, 57] comprising 1598 patients were included. Direct comparisons mainly involved MAKO, HURWA, SkyWalker, YUANHUA, CORI, ROSA, Brainlab Knee, and TiRobot versus C-TKA, with additional comparisons between HURWA and CORI and between HURWA and Brainlab Knee, enabling indirect comparisons among all robotic systems.

For the KSS-Knee score, 19 studies [25, 27, 29, 32–34, 37–44, 47, 48, 52, 54, 58] including 1459 patients were analyzed. Direct comparisons mainly involved MAKO, HURWA, SkyWalker, YUANHUA, CORI, ROSA, Brainlab Knee, and TiRobot versus C-TKA, with additional comparisons between HURWA and CORI and between HURWA and Brainlab Knee, enabling indirect comparisons among all robotic systems.

For the KSS-SF score, 9 studies [25, 27, 32–34, 40, 44, 47, 48] involving 794 patients were included. Direct comparisons mainly involved MAKO, HURWA, SkyWalker, CORI, and ROSA versus C-TKA, with additional comparisons between HURWA and CORI and between HURWA and Brainlab Knee, enabling indirect comparisons across all relevant robotic systems.

**Fig. 3:**
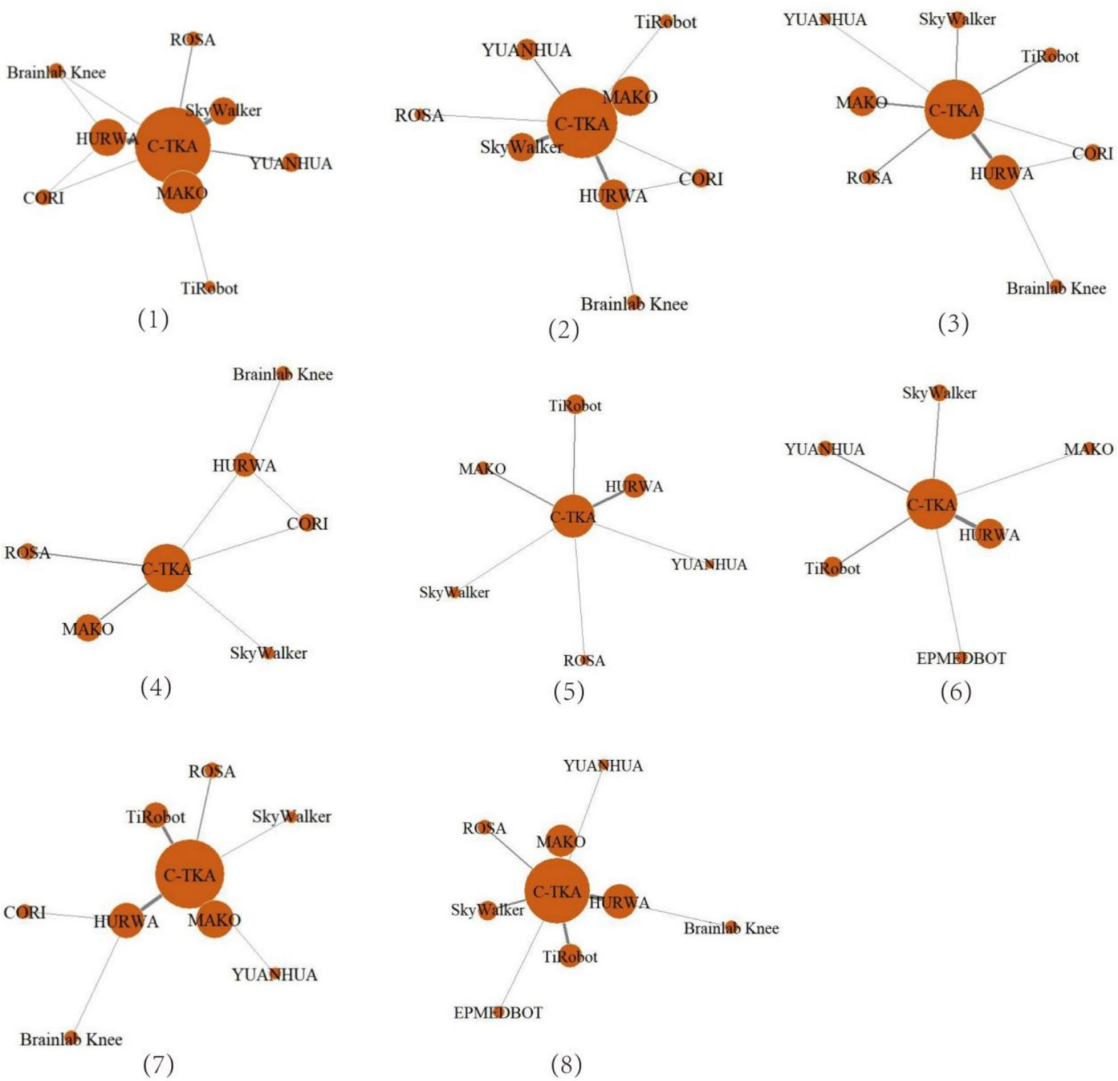
Network plot. Nodes represent different interventions, and lines represent direct comparisons between interventions, with line thickness indicating the number of comparisons. **1** Operative time, **2** blood loss, **3** KSS-knee score, **4** KSS-function score, **5** HSS, **6** WOMAC, **7** ROM, **8** HKA angle deviation. A: C-TKA,B: MAKO, C: HURWA, D: SkyWalker, E: YUANHUA, F: CORI, G: ROSA, H: Brainlab Knee, I: TiRobot, J: EPMEDBOT

For the HSS, 10 studies [27, 28, 30, 34, 38, 40, 50, 55, 58, 60 involving 690 patients were included. For the WOMAC, 13 studies [27, 29, 30, 38–40, 42, 45, 51, 53, 54, 59, 60] comprising 904 patients were analyzed. For postoperative ROM, 19 studies [28, 32–35, 37–41, 43, 44, 47–51, 54, 60] involving 1623 patients were included. For HKA angle deviation, 24 studies [29, 32–35, 37–43, 45, 47, 49–56, 59, 60] including 1745 patients were analyzed. All outcomes enabled direct comparisons between different RA-TKA systems and C-TKA, thereby allowing indirect comparisons across all relevant robotic systems. The network plots are shown in Fig. 3.

### Meta-analysis results

#### Operative time

A total of 31 studies [25–53, 56, 57] reported the operative time. The results demonstrated that C-TKA was associated with a significantly shorter operative time than MAKO (MD = 28, 95% CI = 18, 37), HURWA (MD = 19, 95% CI = 8.8, 30), SkyWalker (MD = 19, 95% CI = 6.5, 31), ROSA (MD = 29, 95% CI = 8.7, 50), and Brainlab Knee (MD = 42, 95% CI = 19, 65). In addition, CORI had a shorter operative time than Brainlab Knee did (MD = 35, 95% CI = 4.6, 65). No significant differences were observed among the remaining comparisons. On the basis of optimal ranking probabilities, C-TKA had the highest probability of being the optimal intervention for operative time (93%), followed by CORI (77%), YUANHUA (62%), TiRobot (61%), SkyWalker (51%), HURWA (49%), MAKO (26%), ROSA (25%), and Brainlab Knee (7%).

#### Blood loss

22 studies [25, 26, 28, 30, 32, 33, 35–38, 40–42, 44, 46, 47, 49–53, 57] reported blood loss. No statistically significant differences were identified in any pairwise comparison. According to the ranking probabilities, SkyWalker had the highest probability of being optimal (87%), followed by HURWA (70%), MAKO (67%), CORI (56%), TiRobot (53%), YUANHUA (49%), C-TKA (40%), ROSA (16%), and Brainlab Knee (13%).

#### KSS-knee score

19 studies [25, 27, 29, 32–34, 37–44, 47, 48, 52, 54, 58] reported KSS knee scores. ROSA achieved significantly higher KSS knee scores than C-TKA (MD = 3.8, 95% CI = 0.14, 7.5), MAKO (MD = 5.5, 95% CI = 1.5, 9.8), HURWA (MD = 4.5, 95% CI = 0.47, 8.6), SkyWalker (MD = 7.4, 95% CI = 3.4, 12), and TiRobot (MD = 5.5, 95% CI = 0.82, 10). In contrast, C-TKA (MD = 3.7, 95% CI = 1.8, 5.6), HURWA (MD = 3.0, 95% CI = 0.37, 5.6), and CORI (MD = 3.7, 95% CI = 0.47, 6.7) demonstrated higher KSS knee scores than did SkyWalker. No other significant differences were observed. According to the optimal ranking probabilities, SkyWalker ranked highest for the KSS knee score (91%), followed by YUANHUA (76%), MAKO (67%), TiRobot (64%), HURWA (48%), Brainlab Knee (36%), CORI (34%), C-TKA (31%), and ROSA (2%).

#### KSS-function score

9 studies [25, 27, 32–34, 40, 44, 47, 48] reported KSS function scores. C-TKA demonstrated significantly higher KSS function scores than HURWA did (MD = 9.4, 95% CI = 1.1–21), while ROSA also outperformed HURWA (MD = 12, 95% CI = 1.2–25). No significant differences were found among the remaining comparisons. HURWA had the highest probability of being optimal (87%), followed by Brainlab Knee (79%), CORI (68%), SkyWalker (57%), C-TKA (24.4%), MAKO (24.3%), and ROSA (11%).

#### HSS

10 studies [27, 28, 30, 34, 38, 40, 50, 55, 58, 60] reported HSS scores. No statistically significant differences were observed in any pairwise comparisons. According to the ranking probabilities, MAKO ranked highest (85%), followed by SkyWalker (65%), TiRobot (60%), HURWA (53%), YUANHUA (43%), C-TKA (30%), and ROSA (13%).

#### WOMAC

13 studies [27, 29, 30, 38–40, 42, 45, 51, 53, 54, 59, 60] reported WOMAC scores. Similarly, no statistically significant differences were identified in any pairwise comparison. The YUANHUA had the highest probability of being optimal (76%), followed by SkyWalker (71%), MAKO (66%), TiRobot (58%), HURWA (32%), C-TKA (31%), and EPMEDBOT (16%).

#### ROMs

19 studies [28, 32–35, 37–41, 43, 44, 47–51, 54, 60] reported postoperative ROM. C-TKA demonstrated a significantly greater ROM than HURWA did (MD = 4.1, 95% CI = 1.1–7.2), while no other significant differences were observed. According to the ranking probabilities, CORI ranked highest (69%), followed by HURWA (68%), ROSA (64%), TiRobot (50%), Brainlab Knee (48.7%), MAKO (48.5%), SkyWalker (46%), YUANHUA (44%), and C-TKA (11%).

#### HKA angle deviation

24 studies [29, 32–35, 37–43, 45, 47, 49–56, 59, 60] reported HKA angle deviation. C-TKA was associated with significantly greater HKA deviation than MAKO (MD = −1.3, 95% CI = −2.0, −0.64), HURWA (MD = −1.1, 95% CI = −1.8, −0.48), SkyWalker (MD = −2.0, 95% CI = −3.1, −0.98), TiRobot (MD = −0.86, 95% CI = −1.8, −0.021), and EPMEDBOT (MD = −2.3, 95% CI = −4.4, −0.26). No significant differences were observed among the remaining comparisons. SkyWalker ranked highest (87%), followed by EPMEDBOT (86%), MAKO (65%), HURWA (55%), YUANHUA (46%), TiRobot (44%), ROSA (38%), Brainlab Knee (20%), and C-TKA (10%) in terms of the optimal ranking probabilities.

**Fig. 4:**
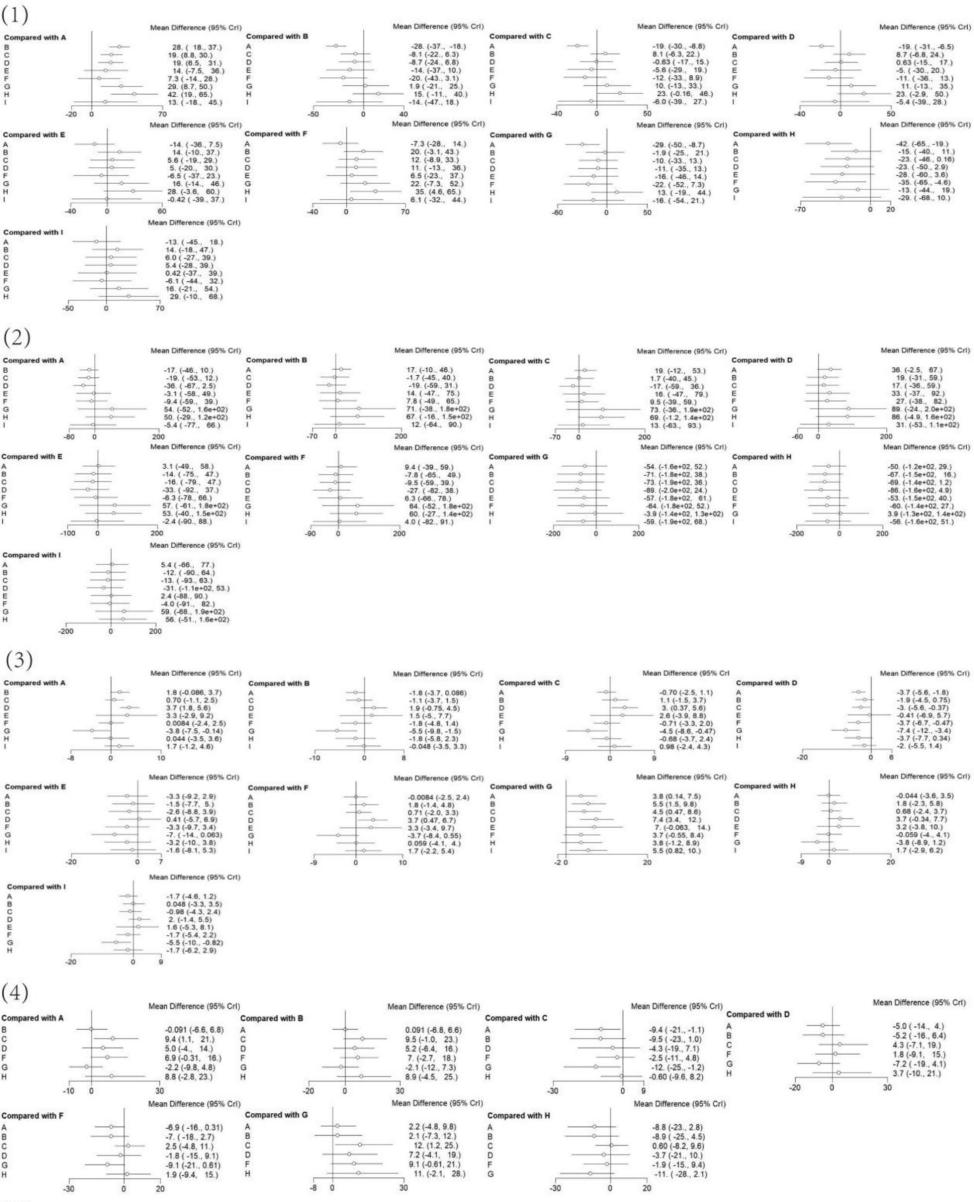

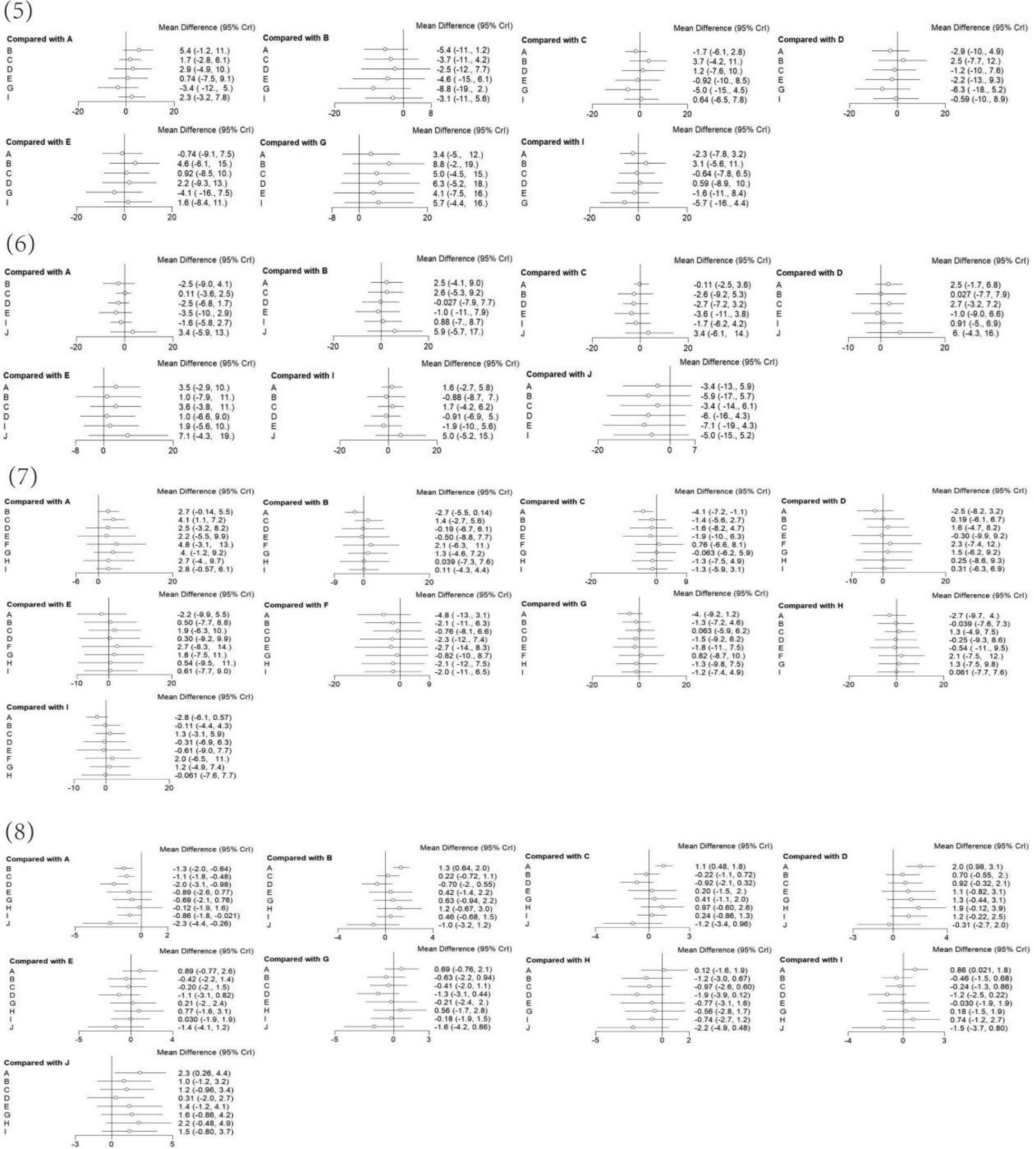
Network meta-analysis forest plot. Points represent the MD for each intervention, and the horizontal lines indicate the 95% CI. For continuous outcomes, when MD is used as the effect size, an MD and its 95% CI entirely below or above zero indicate a statistically significant difference. **1** Operative time, **2** blood loss, **3** KSS-knee score, **4** KSS-function score, **5** HSS, **6** WOMAC, **7** ROM, **8** HKA angle deviation A: C-TKA, B: MAKO, C: HURWA, D: SkyWalker, E: YUANHUA, F: CORI, G: ROSA, H: Brainlab Knee, I: TiRobot, J: EPMEDBOT

**Fig. 5:**
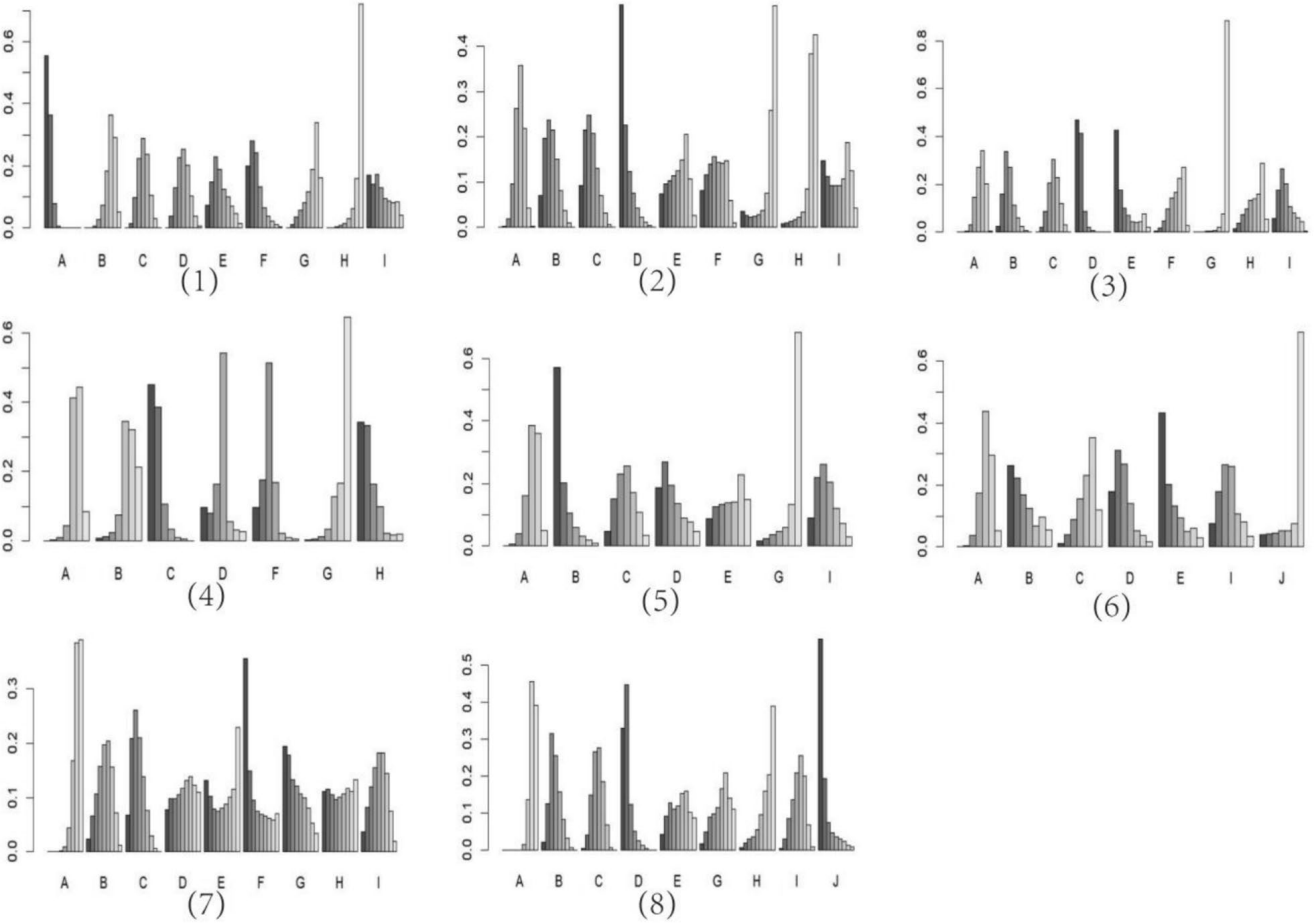
Optimal probability ranking diagram. The horizontal axis represents the interventions, and the vertical axis represents the probability values. The bar chart illustrates the ranking of treatment effects, with darker colors indicating better performance and black indicating the optimal intervention. **1** Operative time, **2** blood loss, **3** KSS-knee score, **4** KSS-function score, **5** HSS, **6** WOMAC, **7** ROM, **8** HKA angle deviation. A: C-TKA, B: MAKO, C: HURWA, D: SkyWalker, E: YUANHUA, F: CORI, G: ROSA, H: Brainlab Knee, I: TiRobot, J: EPMEDBOT

The meta-analysis results are presented in Figs. 4 and 5 and Table 2.

**Table 2:** Comprehensive optimal probability ranking table

	C-TKA	MAKO	HURWA	SkyWalker	YUANHUA	CORI	ROSA	Brainlab knee	TiRobot	EPMEDBOT
Operative Time	0.93270312	0.25896719	0.48797656	0.50772344	0.61747969	0.76553281	0.25210469	0.06518125	0.61233125	–
Blood loss	0.4013313	0.6704031	0.6960875	0.8656734	0.4902922	0.5569172	0.1593688	0.1322719	0.5276547	–
KSS-knee score	0.30794531	0.67202969	0.48485312	0.91310000	0.75783750	0.33838438	0.02340156	0.36004063	0.64240781	–
KSS-function score	0.2439896	0.2425083	0.8678792	0.5680479	–	0.6823521	0.1058021	0.7894208	–	–
HSS score	0.2996833	0.8549083	0.5311813	0.6510458	0.4340229	–	0.1260583	–	0.6031000	–
WOMAC	0.3099604	0.6634687	0.3176521	0.7082146	0.7613917	–	–	–	0.5795167	0.1597958
ROM	0.1126141	0.4852172	0.6840969	0.4620984	0.4393203	0.6857453	0.6427156	0.4868438	0.5013484	–
HKA angle deviation	0.09739688	0.64708594	0.54605313	0.86686250	0.45982500	–	0.38046719	0.19980156	0.43922187	0.86328594

#### Consistency and convergence assessment

**Fig. 6:**
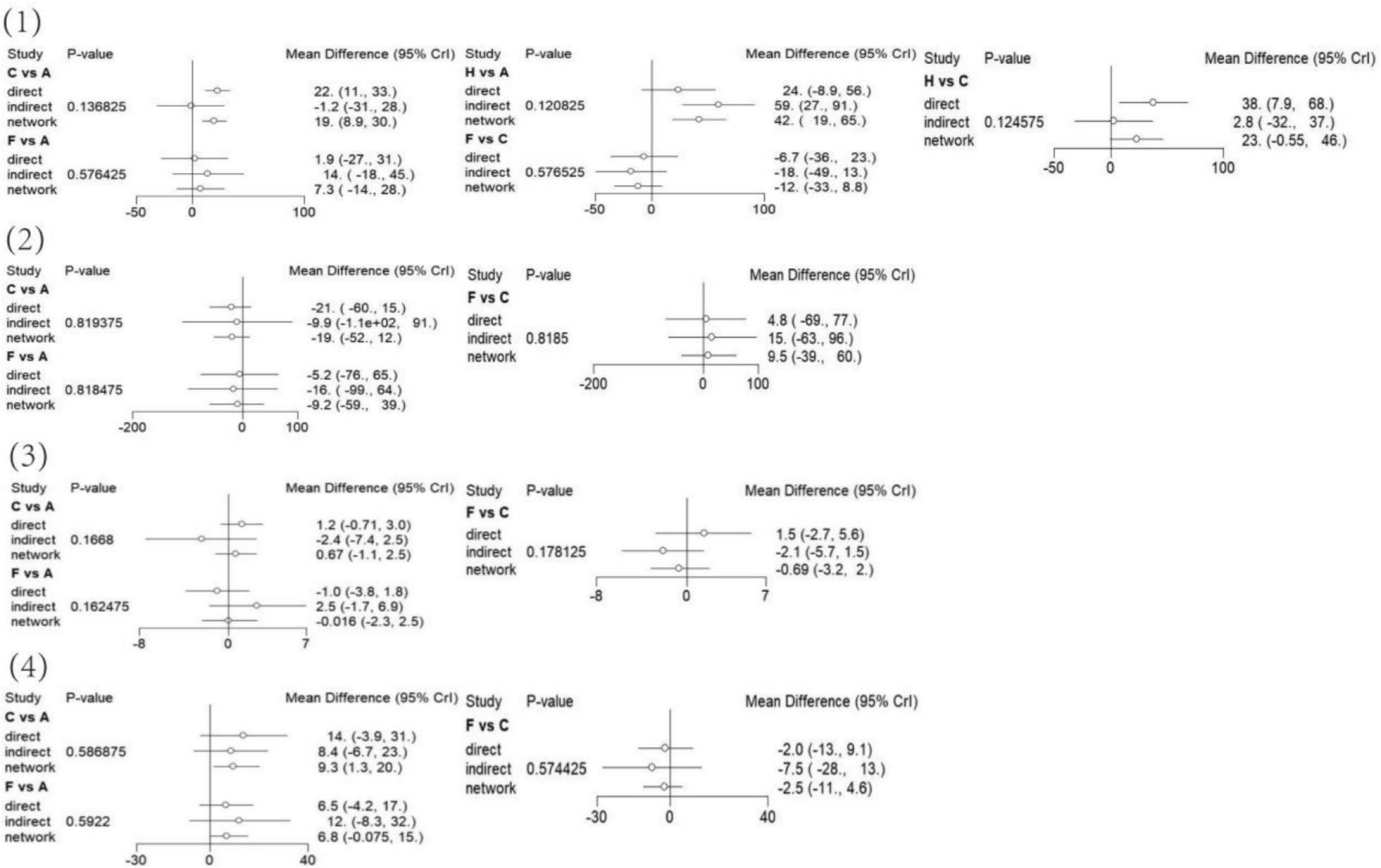
Node-splitting analysis results. **1** Operative time, **2** blood loss, **3** KSS-knee score, **4** KSS-function score, A: C-TKA, C: HURWA, F: CORI, H: Brainlab knee.

Consistency across the network was evaluated via the node-splitting method for the 8 studies. However, only the operative time, blood loss, KSS knee score and KSS function score had sufficient comparative data to assess inconsistency. The results showed that all node-splitting analyses yielded P > 0.05, indicating good network consistency and no evidence of disagreement between direct and indirect estimates (Fig. 6).

**Fig. 7:**
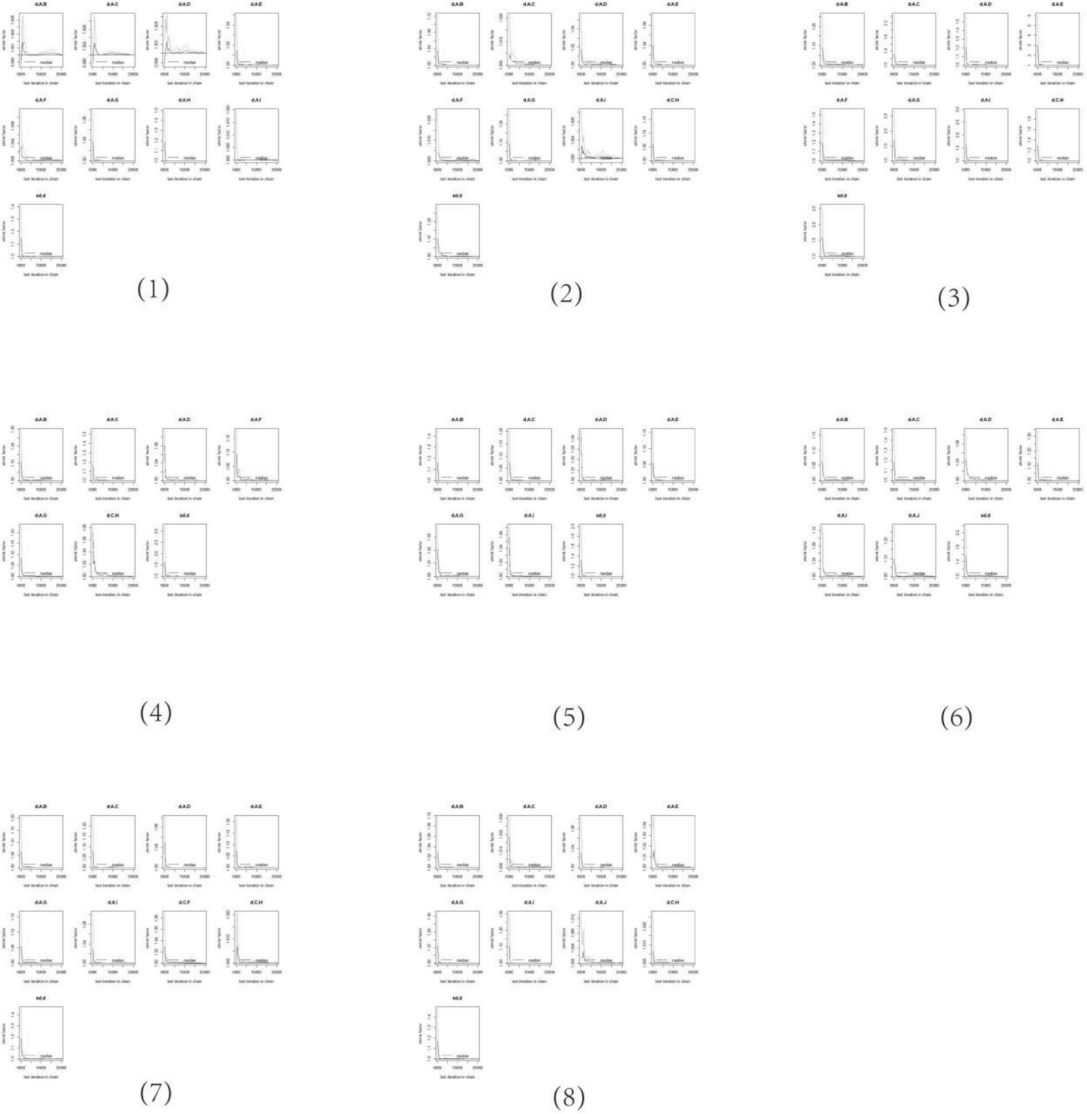
Brooks–Gelman–Rubin diagnostic plot. The horizontal axis represents the number of iterations in the chains, and the vertical axis represents the shrink factor. The solid black line indicates the median shrink factor, whereas the red dashed line represents the 97.5th percentile of the shrink factor. Fewer iterations indicate better convergence. **1** Operative time, **2** blood loss, **3** KSS-knee score, **4** KSS-function score, **5** HSS, **6** WOMAC, **7** ROM, **8** HKA angle deviation

Model convergence was assessed via Brooks–Gelman–Rubin diagnostic plots and the PSRF for all 8 studies. The results demonstrated that both the median shrink factor and the 97.5th percentile of the shrink factor approached 1 and stabilized after 10,000 iterations. All the PSRF values were equal to 1.00. These findings indicate good model convergence and adequate model fit, suggesting that the results of the network meta-analysis are reliable (Fig. 7 and Table 3).

**Table 3:** Potential scale reduction factor

	Point est	Upper C.I
*Operative time*		
d.A.B	1.00	1.00
d.A.C	1.00	1.00
d.A.D	1.00	1.00
d.A.E	1.00	1.00
d.A.F	1.00	1.00
d.A.G	1.00	1.00
d.A.H	1.00	1.00
d.A.I	1.00	1.00
SD.d	1.00	1.00
*Blood loss*		
d.A.B	1.00	1.00
d.A.C	1.00	1.00
d.A.D	1.00	1.00
d.A.E	1.00	1.00
d.A.F	1.00	1.00
d.A.G	1.00	1.00
d.A.I	1.00	1.00
d.C.H	1.00	1.00
SD.d	1.00	1.00
KSS-*knee score*		
d.A.B	1.00	1.00
d.A.C	1.00	1.00
d.A.D	1.00	1.00
d.A.E	1.00	1.00
d.A.F	1.00	1.00
d.A.G	1.00	1.00
d.A.I	1.00	1.00
d.C.H	1.00	1.00
SD.d	1.00	1.00
KSS-*function score*		
d.A.B	1.00	1.00
d.A.C	1.00	1.00
d.A.D	1.00	1.00
d.A.F	1.00	1.00
d.A.G	1.00	1.00
d.C.H	1.00	1.00
SD.d	1.00	1.00
HSS *scor*e		
d.A.B	1.00	1.00
d.A.C	1.00	1.00
d.A.D	1.00	1.00
d.A.E	1.00	1.00
d.A.G	1.00	1.00
d.A.I	1.00	1.00
SD.d	1.00	1.00
WOMAC		
d.A.B	1.00	1.00
d.A.C	1.00	1.00
d.A.D	1.00	1.00
d.A.E	1.00	1.00
d.A.I	1.00	1.00
d.A.J	1.00	1.00
SD.d	1.00	1.00
ROM		
d.A.B	1.00	1.00
d.A.C	1.00	1.00
d.A.D	1.00	1.00
d.A.E	1.00	1.00
d.A.G	1.00	1.00
d.A.I	1.00	1.00
d.C.F	1.00	1.00
d.C.H	1.00	1.00
SD.d	1.00	1.00
HKA *angle deviation*		
d.A.B	1.00	1.00
d.A.C	1.00	1.00
d.A.D	1.00	1.00
d.A.E	1.00	1.00
d.A.G	1.00	1.00
d.A.I	1.00	1.00
d.A.J	1.00	1.00
d.C.H	1.00	1.00
SD.d	1.00	1.00

## Discussion

To our knowledge, previous studies have focused predominantly on meta-analyses and clinical comparisons between RA-TKA and C-TKA, whereas systematic comparisons of the effectiveness and safety of different RA-TKA systems remain limited. With the rapid advancement of robotic technology in orthopedics, RA-TKA has increasingly emerged as an important direction in the evolution of total knee arthroplasty. However, owing to heterogeneity in robotic platforms, navigation strategies, and execution mechanisms, surgical performance and clinical outcomes may vary across different robotic systems. In this study, we constructed an evidence network incorporating both RA-TKA and C-TKA, enabling indirect comparisons of the safety and effectiveness of 9 RA-TKA procedures in addition to direct comparisons between RA-TKA and C-TKA. The primary aim was to clarify the relative advantages of individual RA-TKA systems in terms of safety and effectiveness, as well as their differences compared with C-TKA. These findings may assist surgeons in selecting appropriate surgical strategies and help identify current limitations of RA-TKA, thereby informing future optimization.

With respect to operative time, the probability ranking results demonstrated that C-TKA was significantly superior to MAKO, HURWA, SkyWalker, ROSA, and Brainlab Knee in pairwise comparisons. China has experienced a substantial increase in the volume of knee arthroplasty procedures. Previous studies reported that the annual number of TKAs in China increased from 2011–2019, representing a 5.9-fold increase [61]. C-TKA has been proven to provide reliable long-term outcomes, and surgeons’ high level of proficiency and ability to manage complex intraoperative scenarios play crucial roles in reducing the operative time. In contrast, orthopedic robotic-assisted technologies are still in a developmental stage. For example, although the MAKO system received FDA approval for UKA, TKA, and THA in 2005, ROSA was not approved until 2019 [13]. Most robotic platforms continue to undergo iterative development, and system optimization and technical maturity have not yet been fully achieved. Moreover, RA-TKA generally requires additional time for system setup, intraoperative execution, and postoperative dismantling, all of which may collectively contribute to prolonged operative time [62].

Intraoperative blood loss is a key indicator of surgical safety. In the optimal probability ranking, the SkyWalker method demonstrated the highest likelihood of being the optimal option, followed by HURWA. Previous studies have reported that although RA-TKA is often associated with a longer operative time, intraoperative blood loss does not significantly differ from that of C-TKA, and a prolonged operative time does not necessarily translate into increased blood loss [63]. While perioperative hemostatic management and tourniquet use may influence blood loss, the incision technique and intraoperative precision are likely more critical determinants. The SkyWalker offers millimeter-level osteotomy accuracy and precise lower limb alignment control. Its nonintramedullary positioning design avoids violations of the medullary canal and reduces cancellous bone bleeding. In addition, robotic-arm–assisted precision minimizes unnecessary soft tissue dissection and vascular injury, thereby reducing intraoperative blood loss and postoperative tissue trauma [15]. Consequently, the SkyWalker may confer a potential advantage in controlling intraoperative blood loss.

The KSS-Knee score primarily emphasizes postoperative pain while also reflecting joint mobility and stability. The probability ranking indicated that the SkyWalker algorithm was most likely to achieve the optimal outcome, followed by the YUANHUA. By minimizing unnecessary periarticular soft tissue release and bone injury, the SkyWalker may effectively reduce postoperative inflammatory responses. Its nonintramedullary technique avoids medullary canal irritation–related deep pain and may reduce chronic pain associated with prosthetic micromotion and abnormal soft tissue tension. Furthermore, its minimally invasive surgical strategy may shorten the duration of postoperative inflammatory responses [64]. Therefore, the SkyWalker may offer advantages in improving postoperative pain outcomes following TKA.

The KSS function score primarily reflects patients’ postoperative walking ability and stair-climbing performance. In this study, the probability ranking showed that the HURWA had the highest likelihood of achieving the optimal outcome, followed by Brainlab Knee. Through preoperative three-dimensional visualization planning and high-precision robotic execution, HURWA can significantly improve the accuracy of lower limb alignment reconstruction and prosthesis positioning. This provides a stable biomechanical foundation for postoperative functional recovery, leading to greater improvements in mobility, stability, and walking-related functional domains of the knee [65].

The HSS score comprehensively reflects pain, function, range of motion, muscle strength, flexion deformity, and joint stability. The probability ranking indicated that MAKO had the highest likelihood of being optimal, followed by SkyWalker. The AccuStop haptic feedback technology used by MAKO enables the precise control of osteotomy angles, positions, and implant sizing, which may help preserve soft tissues and healthy bone during surgery, thereby reducing postoperative pain and swelling and facilitating functional recovery [66]. This may explain its potential advantage in HSS outcomes. Additionally, in conjunction with the KSS knee score findings, the SkyWalker may demonstrate more consistent benefits in postoperative pain relief.

The WOMAC score reflects pain, stiffness, and physical function and is a key outcome measure reflecting postoperative functional recovery and quality-of-life improvement after TKA [67]. The probability ranking suggested that the YUANHUA had the highest likelihood of achieving optimal outcomes, followed by the SkyWalker. By assisting osteotomy within predefined safe zones, the YUANHUA may reduce the risk of excessive bone resection and periarticular ligament injury, thereby alleviating postoperative pain and stiffness and promoting functional recovery [68]. Thus, the YUANHUA may offer certain advantages in TKA for patients with knee osteoarthritis, whereas the SkyWalker also demonstrated favorable overall performance.

The ROM is a core component of multiple knee function scoring systems, reflecting flexion and extension capacity. In probability ranking, the CORI exhibited the highest likelihood of being optimal, followed by HURWA. CORI allows real-time intraoperative adjustments under image guidance and repeated validation of flexion–extension gaps before final implant fixation. This approach may facilitate greater postoperative ROM, particularly during early rehabilitation [7]. Therefore, CORI may be advantageous for patients in whom postoperative flexion–extension improvement is a primary goal. Moreover, considering its performance in terms of functional outcomes, HURWA also demonstrated potential benefits in functional recovery.

The HKA angle deviation is defined as the deviation of the hip–knee–ankle angle from the neutral 180° alignment and is used to quantitatively assess varus or valgus deviations of the lower limb mechanical axis after TKA [69]. In probability ranking, the SkyWalker method demonstrated the highest likelihood of achieving optimal alignment, followed by the EPMEDBOT method. By integrating preoperative CT-based three-dimensional planning with precise intraoperative robotic execution, the SkyWalker enables personalized lower limb alignment reconstruction and provides accurate technical support for deformity correction. This may reduce postoperative HKA deviation, improve the accuracy and consistency of mechanical axis restoration, and potentially lower the risk of long-term implant loosening [70]. Therefore, for patients in whom correction of lower limb deformity is a primary surgical objective, the SkyWalker may offer a distinct advantage.

## Conclusions

This study systematically evaluated multiple clinical outcomes of RA-TKA through a network meta-analysis. Overall, RA-TKA demonstrated superior clinical effectiveness compared with C-TKA; however, individual robotic systems presented distinct advantages across specific outcome measures. Among the evaluated platforms, SkyWalker and HURWA showed relatively superior overall performance.

In terms of pain relief, the SkyWalker system may offer particular advantages. For postoperative knee function recovery, HURWA demonstrated more favorable outcomes. MAKO may be more suitable for patients whose primary clinical features include pain accompanied by functional limitations, deformities, and reduced muscle strength. For patients diagnosed with knee osteoarthritis, the YUANHUA may provide benefits in terms of functional improvement and symptom relief. In contrast, for patients whose correction of lower limb deformity and restoration of mechanical alignment are primary surgical objectives, the SkyWalker may represent a better choice. For patients with poorer baseline characteristics or those requiring a shorter operative time, C-TKA remains a clinically valuable option.

No large-scale intraoperative or postoperative serious complications associated with RA-TKA were observed in this study, suggesting that RA-TKA is safe during either the perioperative or postoperative period. Although RA-TKA did not demonstrate a clear advantage in reducing the operative time or improving surgical efficiency—likely owing to the complexity of robotic workflows and their reliance on high-precision execution—this represents an important area for future optimization. Nevertheless, RA-TKA continues to show substantial clinical potential in enhancing postoperative functional recovery and achieving accurate lower limb alignment reconstruction.

## Limitations

(1) As an emerging clinical technology, the number of published comparative studies on RA-TKA remains limited, which restricts the inclusion of a larger body of evidence in the present analysis.

(2) In this study, several included publications did not report the operative time, intraoperative blood loss, KSS knee score, or ROM; therefore, the EPMEDBOT could not be included in these four analyses. Similarly, EPMEDBOT, YUANHUA, and TiRobot were not included in the analysis of the KSS function score. CORI, Brainlab Knee, and EPMEDBOT were not included in the HSS score analysis, whereas CORI, ROSA, and Brainlab Knee were not included in the WOMAC score analysis. In addition, the CORI was not included in the analysis of HKA angle deviation.

(3) Most of the included studies did not clearly describe the methods of allocation or allocation concealment, which may have introduced selection bias and information bias.

(4) Most studies lacked high-quality long-term follow-up data on safety and effectiveness outcomes, such as prosthesis loosening and revision rates, thereby limiting the robustness and generalizability of the conclusions regarding RA-TKA.

(5) The learning curve associated with robotic surgery, differences in cost-effectiveness, and the applicability of robotic systems to complex cases were not adequately adjusted for or stratified in most studies.

Explanation of these limitations:

(1)(2) Orthopedic robotic technology has a relatively short history of development, with most robot-assisted systems being introduced into clinical practice only within the past two decades. Consequently, the available literature remains limited, and some original studies did not report certain outcome measures. Future studies incorporating a larger number of publications are needed to improve the completeness of outcome assessments.

(3) This limitation may be attributable to methodological shortcomings in the original studies, issues related to reporting quality, or the retrospective nature of some studies, which may not be suitable for randomization, blinding, or allocation concealment.

(4)(5) Future research should prioritize multicenter, large-sample randomized controlled trials with standardized technical protocols and extended follow-up periods to confirm the long-term safety and effectiveness of robotic technologies. Moreover, standardized reporting of stratified analyses for complex cases and cost-related data is warranted to reduce bias and improve the overall quality of evidence.

## Supplementary Information

Below is the link to the electronic supplementary material.

Supplementary file1 (DOCX 40 KB)

## Data Availability

All analyses and original data of this study are included in the article or supplementary materials.

## Abbreviations

TKA: Total knee arthroplasty
KOA: Knee osteoarthritis
RA: Rheumatoid arthritis
C-TKA: Conventional total knee arthroplasty
THA: Total hip arthroplasty
RA-TKA: Robot-assisted total knee arthroplasty
MAKO: MAKO robot-assisted total knee arthroplasty
CORI: CORI robot-assisted total knee arthroplasty
ROSA: ROSA robot-assisted total knee arthroplasty
Brainlab Knee: Brainlab knee robot-assisted total knee arthroplasty
EPMEDBOT: EPMEDBOT robot-assisted total knee arthroplasty
SkyWalker: SkyWalker robot-assisted total knee arthroplasty
TiRobot: TiRobot robot-assisted total knee arthroplasty
HURWA: HURWA robot-assisted total knee arthroplasty
YUANHUA: YUANHUA robot-assisted total knee arthroplasty
CBM: Chinese biomedical literature database
CNKI: Chinese national knowledge infrastructure
MD: Mean difference
CI: Confidence intervals
PSRF: Potential scale reduction factor
FDA: Food and Drug Administration
UKA: Unicompartmental knee arthroplasty
HSS: Hospital for Special Surgery Knee Score
WOMAC: The Western Ontario and McMaster Universities Osteoarthritis Index
ROMs: Range of motion
HKA: Hip knee ankle
