# Supplementary material for: Safety and efficacy of robot-assisted total knee arthroplasty: a network meta-analysis and systematic review
Source: J Orthop Surg Res. 2026 May 2;21:377. doi: 10.1186/s13018-026-06889-y (PMC13343880; doi:10.1186/s13018-026-06889-y)
Supplement: Supplementary file 1 — Supplementary file1 (DOCX 40 KB) [file 13018_2026_6889_MOESM1_ESM.docx]

| **Section and Topic** | **Item #** | **Checklist item** | **Location where item is reported** |
| --- | --- | --- | --- |
| **TITLE** | | |  |
| Title | 1 | Safety and Efficacy of Robot-Assisted Total Knee Arthroplasty: A Network Meta-Analysis and Systematic Review |  |
| **ABSTRACT** | | |  |
| Abstract | 2 | Background In the context of the aging of the global population, the prevalence of knee joint disorders continues to rise. Concurrently, the integration of robotic systems and intelligent implants represents an inevitable trend in orthopedic surgery. A comprehensive evaluation of the safety and effectiveness of robot-assisted total knee arthroplasty (RA-TKA) is therefore urgently needed to inform clinical decision-making.  Objective To explore the advantages of 9 RA-TKAs across 8 outcomes.  Methods A systematic literature search was conducted in the PubMed, Web of Science, Embase, Cochrane Library, CBM, CNKI, Wanfang, and VIP databases from inception to December 1, 2025. The risk of bias and methodological quality were assessed via Review Manager (version 5.4). Network meta-analysis was performed via RStudio (version 4.4.1).  Results A total of 36 studies involving 2,841 patients were included. In direct comparisons, conventional TKA (C-TKA) yielded shorter operative times than MAKO, HURWA, SkyWalker, ROSA, and Brainlab Knee did. CORI also had a shorter operative time than Brainlab Knee did. Compared with the C-TKA, MAKO, HURWA, SkyWalker and TiRobot groups, the ROSA group presented higher KSS-knee scores. In addition, C-TKA, HURWA, and CORI presented higher KSS-knee scores than did SkyWalker. For the KSS-function scores, the C-TKA and ROSA scores were higher than the HURWA score. C-TKA demonstrated a greater postoperative ROM than HURWA did. For HKA angle deviation, C-TKA resulted in greater deviation than MAKO, HURWA, SkyWalker, TiRobot, and EPMEDBOT did. In the comprehensive best probability ranking, C-TKA (93%) ranked highest in terms of operative time. SkyWalker (87%) ranked highest in terms of blood loss. SkyWalker (91%) ranked highest in terms of the KSS-knee scores. HURWA (87%) ranked highest in terms of the KSS function scores. MAKO (85%) ranked highest for HSS. The YUANHUA (76%) ranked highest for the WOMAC. The CORI (69%) ranked highest for ROM. SkyWalker (87%) ranked highest for HKA angle deviation.  Conclusions Overall, RA-TKA demonstrated superior safety and effectiveness compared with C-TKA, with different robotic systems exhibiting distinct advantages across outcome measures. Nevertheless, C-TKA retains a significant advantage in reducing the operative time, highlighting an important area for further optimization of robotic-assisted TKA.  Keywords: robot-assisted, total knee arthroplasty, network meta-analysis, systematic review |  |
| **INTRODUCTION** | | |  |
| Rationale | 3 | As far as we know, there have been very few studies on this kind before. The comparative advantages of different RA-TKA systems remain unclear. |  |
| Objectives | 4 | To evaluate the comparative advantages of different RA-TKA systems, this study aims to systematically assess the safety and effectiveness of the MAKO, HURWA, SkyWalker, Yuanhua, CORI, ROSA, Brainlab Knee, TiRobot, and EPMEDBOT systems via network meta-analysis. |  |
| **METHODS** | | |  |
| Eligibility criteria | 5 | (1) randomized controlled trials (RCTs) or retrospective cohort studies (RCs); (2) studies involving patients undergoing TKA; (3) studies reporting at least one of the following outcomes: operative time, blood loss, Knee Society score (KSS) - knee score, KSS - function score, Hospital for Special Surgery (HSS) score, Western Ontario and McMaster Universities Osteoarthritis Index (WOMAC) score, range of motion (ROM), and hip–knee–ankle (HKA) angle deviation; and (4) robot-assisted procedures involving preoperative planning, intraoperative positioning, bone resection, trial implantation, and prosthesis implantation. |  |
| Information sources | 6 | A comprehensive literature search was conducted in the electronic databases PubMed, Web of Science, Embase, the Cochrane Library, CBM, CNKI, Wanfang, and VIP from inception to December 1, 2025. |  |
| Search strategy | 7 | The search strategy used the keywords robot-assisted, total knee arthroplasty, randomized, random, and comparison, which were searched in all fields. |  |
| Selection process | 8 | Two researchers (Zhenhua Wu and Han Li) independently screened and verified the eligible studies, with disagreements adjudicated by a third researcher (Jixiong Yong). The screening process included the removal of duplicate records, initial screening of titles, evaluation of abstracts and keywords, and full-text review to ensure data completeness. |  |
| Data collection process | 9 | Two researchers (Zhenhua Wu and Han Li) independently screened and verified the eligible studies, with disagreements adjudicated by a third researcher (Jixiong Yong). The screening process included the removal of duplicate records, initial screening of titles, evaluation of abstracts and keywords, and full-text review to ensure data completeness. The following information was extracted: first author, year of publication, country, sample size (male/female), mean age, follow-up duration, intervention details, and outcome measures. |  |
| Data items | 10a | operative time, blood loss, Knee Society score (KSS) - knee score, KSS - function score, Hospital for Special Surgery (HSS) score, Western Ontario and McMaster Universities Osteoarthritis Index (WOMAC) score, range of motion (ROM), and hip–knee–ankle (HKA) angle deviation |  |
|  | 10b | first author, year of publication, country, sample size (male/female), mean age, follow-up duration, intervention details |  |
| Study risk of bias assessment | 11 | The risk of bias in the included studies was assessed via Review Manager 5.4. evaluating random sequence generation, allocation concealment, blinding, completeness of outcome data, selective reporting, and other potential sources of bias. 2 |  |
| Effect measures | 12 | All outcomes were treated as continuous variables and analyzed via mean differences (MDs) with corresponding 95% confidence intervals. |  |
| Synthesis methods | 13a | Studies were grouped for synthesis according to the type of robotic-assisted total knee arthroplasty and conventional TKA. Only randomized controlled trials and comparative cohort studies reporting at least one predefined outcome were included in the corresponding network meta-analysis. Studies with comparable populations, interventions, and outcome definitions were considered eligible for quantitative synthesis. |  |
|  | 13b | All outcomes were expressed as means and standard deviations. |  |
|  | 13c | Network plots were used to illustrate the geometry of the comparisons, forest plots were employed to present the results of direct comparisons, and optimal ranking probabilities plots and tables were used to depict the treatment ranking of the interventions. |  |
|  | 13d | Network meta-analysis was performed using RStudio (version 4.4.1) with the **“gemtc”** package, and a **random-effects model** was applied. Bayesian Markov chain Monte Carlo (MCMC) simulations were conducted via **JAGS** using the **“rjags”** package. Four Markov chains were run with initial values set at 2.5, a burn-in period of 5,000 iterations, and a total of 10,000 iterations for parameter estimation. Statistical heterogeneity across studies was assessed using the **I² statistic**, with values of **0–25%, 25–75%, and 75–100% indicating low, moderate, and high heterogeneity**, respectively. |  |
|  | 13e | We used subgroup analysis and consistency test to explore the causes of heterogeneity. |  |
|  | 13f | Model convergence was assessed via Brooks–Gelman–Rubin diagnostic plots and the potential scale reduction factor (PSRF). Consistency within closed loops of the network was evaluated via the node-splitting method |  |
| Reporting bias assessment | 14 | We used publication bias funnel to evaluate the stability of the study for more than 10 studies. |  |
| Certainty assessment | 15 | Not applicable. |  |
| **RESULTS** | | |  |
| Study selection | 16a | A total of 372 studies were initially identified through the literature search. After the removal of duplicate records, screening of titles and abstracts, and full-text assessment for eligibility, 36 studies met the inclusion criteria and were included in the network meta-analysis. |  |
|  | 16b | Non-RCT, RS. RA-TKA vs NA-TKA. Intervention and control groups used the same robotic system. |  |
| Study characteristics | 17 | The basic characteristics of the included studies included the first author, year of publication, country, sample size (male/female), mean age, interventions, and outcomes. One study included 2 comparison groups. |  |
| Risk of bias in studies | 18 | In the risk of bias assessment, only 1 study reported the absence of blinding and was therefore judged to be at high risk of bias. The remaining studies were assessed as having a low or moderate risk of bias. Two studies reported the use of envelope randomization with allocation concealment, and 2 studies employed a single-blind design. |  |
| Results of individual studies | 19 | Not applicable. |  |
| Results of syntheses | 20a | For each outcome, network meta-analyses included randomized controlled trials and comparative cohort studies involving different RA-TKA systems and conventional TKA. Overall, the included studies were of moderate to high methodological quality. Most trials reported randomization and outcome assessment, although blinding was generally not feasible due to the nature of surgical interventions. The overall risk of bias across studies was considered low to moderate. |  |
|  | 20b | Operative Time  A total of 31 studies reported the operative time. The results demonstrated that C-TKA was associated with a significantly shorter operative time than MAKO (MD = 28, 95% CI = 18, 37), HURWA (MD = 19, 95% CI = 8.8, 30), SkyWalker (MD = 19, 95% CI = 6.5, 31), ROSA (MD = 29, 95% CI = 8.7, 50), and Brainlab Knee (MD = 42, 95% CI = 19, 65). In addition, CORI had a shorter operative time than Brainlab Knee did (MD = 35, 95% CI = 4.6, 65). No significant differences were observed among the remaining comparisons. On the basis of optimal ranking probabilities, C-TKA had the highest probability of being the optimal intervention for operative time (93%), followed by CORI (77%), YUANHUA (62%), TiRobot (61%), SkyWalker (51%), HURWA (49%), MAKO (26%), ROSA (25%), and Brainlab Knee (7%).  Blood Loss  22 studies reported blood loss. No statistically significant differences were identified in any pairwise comparison. According to the ranking probabilities, SkyWalker had the highest probability of being optimal (87%), followed by HURWA (70%), MAKO (67%), CORI (56%), TiRobot (53%), YUANHUA (49%), C-TKA (40%), ROSA (16%), and Brainlab Knee (13%).  KSS - Knee score  19 studies reported KSS knee scores. ROSA achieved significantly higher KSS knee scores than C-TKA (MD = 3.8, 95% CI = 0.14, 7.5), MAKO (MD = 5.5, 95% CI = 1.5, 9.8), HURWA (MD = 4.5, 95% CI = 0.47, 8.6), SkyWalker (MD = 7.4, 95% CI = 3.4, 12), and TiRobot (MD = 5.5, 95% CI = 0.82, 10). In contrast, C-TKA (MD = 3.7, 95% CI = 1.8, 5.6), HURWA (MD = 3.0, 95% CI = 0.37, 5.6), and CORI (MD = 3.7, 95% CI = 0.47, 6.7) demonstrated higher KSS knee scores than did SkyWalker. No other significant differences were observed. According to the optimal ranking probabilities, SkyWalker ranked highest for the KSS knee score (91%), followed by YUANHUA (76%), MAKO (67%), TiRobot (64%), HURWA (48%), Brainlab Knee (36%), CORI (34%), C-TKA (31%), and ROSA (2%).  KSS - Function score  9 studies reported KSS function scores. C-TKA demonstrated significantly higher KSS function scores than HURWA did (MD = 9.4, 95% CI = 1.1--21), while ROSA also outperformed HURWA (MD = 12, 95% CI = 1.2--25). No significant differences were found among the remaining comparisons. HURWA had the highest probability of being optimal (87%), followed by Brainlab Knee (79%), CORI (68%), SkyWalker (57%), C-TKA (24.4%), MAKO (24.3%), and ROSA (11%).  HSS  10 studies reported HSS scores. No statistically significant differences were observed in any pairwise comparisons. According to the ranking probabilities, MAKO ranked highest (85%), followed by SkyWalker (65%), TiRobot (60%), HURWA (53%), YUANHUA (43%), C-TKA (30%), and ROSA (13%).  WOMAC  13 studies reported WOMAC scores. Similarly, no statistically significant differences were identified in any pairwise comparison. The YUANHUA had the highest probability of being optimal (76%), followed by SkyWalker (71%), MAKO (66%), TiRobot (58%), HURWA (32%), C-TKA (31%), and EPMEDBOT (16%).  ROMs  19 studies reported postoperative ROM. C-TKA demonstrated a significantly greater ROM than HURWA did (MD = 4.1, 95% CI = 1.1–7.2), while no other significant differences were observed. According to the ranking probabilities, CORI ranked highest (69%), followed by HURWA (68%), ROSA (64%), TiRobot (50%), Brainlab Knee (48.7%), MAKO (48.5%), SkyWalker (46%), YUANHUA (44%), and C-TKA (11%).  HKA Angle Deviation  24 studies reported HKA angle deviation. C-TKA was associated with significantly greater HKA deviation than MAKO (MD = -1.3, 95% CI = -2.0, -0.64), HURWA (MD = -1.1, 95% CI = -1.8, -0.48), SkyWalker (MD = -2.0, 95% CI = -3.1, -0.98), TiRobot (MD = -0.86, 95% CI = -1.8, -0.021), and EPMEDBOT (MD = -2.3, 95% CI = -4.4, -0.26). No significant differences were observed among the remaining comparisons. SkyWalker ranked highest (87%), followed by EPMEDBOT (86%), MAKO (65%), HURWA (55%), YUANHUA (46%), TiRobot (44%), ROSA (38%), Brainlab Knee (20%), and C-TKA (10%) in terms of the optimal ranking probabilities. |  |
|  | 20c | Consistency across the network was evaluated via the node-splitting method for the 8 studies. However, only the operative time, blood loss, KSS knee score and KSS function score had sufficient comparative data to assess inconsistency. The results showed that all node-splitting analyses yielded P > 0.05, indicating good network consistency and no evidence of disagreement between direct and indirect estimates. |  |
|  | 20d | Model convergence was assessed via Brooks–Gelman–Rubin diagnostic plots and the PSRF for all 8 studies. The results demonstrated that both the median shrink factor and the 97.5th percentile of the shrink factor approached 1 and stabilized after 10,000 iterations. All the PSRF values were equal to 1.00. These findings indicate good model convergence and adequate model fit, suggesting that the results of the network meta-analysis are reliable (Figure 7 and Table 3). |  |
| Reporting biases | 21 | Publication bias was evaluated for operative time, intraoperative blood loss, KSS knee score, HSS score, postoperative ROM, WOMAC score, and HKA angle deviation. The analysis demonstrated that **only HKA angle deviation exhibited statistically significant asymmetry, suggesting potential publication bias (P = 0.02), while no significant publication bias was observed for the other outcomes (P > 0.05).** |  |
| Certainty of evidence | 22 | Not applicable. |  |
| **DISCUSSION** | | |  |
| Discussion | 23a | The top-ranking RA-TKA systems across the eight outcome measures were discussed in terms of their unique advantages to support our findings. |  |
|  | 23b | In this study, several included publications did not report the operative time, intraoperative blood loss, KSS knee score, or ROM; therefore, the EPMEDBOT could not be included in these four analyses. Similarly, EPMEDBOT, YUANHUA, and TiRobot were not included in the analysis of the KSS function score. CORI, Brainlab Knee, and EPMEDBOT were not included in the HSS score analysis, whereas CORI, ROSA, and Brainlab Knee were not included in the WOMAC score analysis. In addition, the CORI was not included in the analysis of HKA angle deviation.  Most of the included studies did not clearly describe the methods of allocation or allocation concealment, which may have introduced selection bias and information bias.  Most studies lacked high-quality long-term follow-up data on safety and effectiveness outcomes, such as prosthesis loosening and revision rates, thereby limiting the robustness and generalizability of the conclusions regarding RA-TKA.  The learning curve associated with robotic surgery, differences in cost-effectiveness, and the applicability of robotic systems to complex cases were not adequately adjusted for or stratified in most studies. |  |
|  | 23C | As an emerging clinical technology, the number of published comparative studies on RA-TKA remains limited, which restricts the inclusion of a larger body of evidence in the present analysis. |  |
|  | 23d | In this study, we constructed an evidence network incorporating both RA-TKA and C-TKA, enabling indirect comparisons of the safety and effectiveness of **9 RA-TKA** procedures in addition to direct comparisons between RA-TKA and C-TKA. The primary aim was to clarify the relative advantages of individual RA-TKA systems in terms of safety and effectiveness, as well as their differences compared with C-TKA. These findings may assist surgeons in selecting appropriate surgical strategies and help identify current limitations of RA-TKA, thereby informing future optimization. |  |
| **OTHER INFORMATION** | | |  |
| Registration and protocol | 24a | The review was not registered.. |  |
|  | 24b | A protocol was not prepared. |  |
|  | 24c | This study did not cover this aspect. |  |
| Support | 25 | No funding is required for this study. |  |
| Competing interests | 26 | The authors declare that there are no conflicts of interest associated with this study. |  |
| Availability of data, code and other materials | 27 | All analyses and original data of this study are included in the article or supplementary materials. |  |

*From:*  Page MJ, McKenzie JE, Bossuyt PM, Boutron I, Hoffmann TC, Mulrow CD, et al. The PRISMA 2020 statement: an updated guideline for reporting systematic reviews. BMJ 2021;372:n71. doi: 10.1136/bmj.n71
